# A socio-legal inquiry through awards: the “China model” of positive youth development

**DOI:** 10.3389/fpsyg.2026.1811424

**Published:** 2026-04-10

**Authors:** Wenjie Qian

**Affiliations:** Shen Junru Law School, Hangzhou Normal University, Hangzhou, China

**Keywords:** a socio-legal inquiry, China Youth May Fourth Medal, content analysis, growth trajectory, mixed-methods, positive youth development

## Abstract

**Background:**

The China Youth May Fourth Medal serves as a state-endorsed mechanism for modeling positive youth development (PYD). This study aims to decode the archetype of exemplary youth constructed by this socio-legal institution within China’s unique context, moving beyond a direct application of Western PYD models.

**Methods:**

We conducted a socio-legal inquiry employing mixed-methods analysis. A dataset of 1,219 Medal recipients (1997–2023) was examined, focusing on demographic data, career paths, and commendation texts. The study also integrated analysis of extreme counter-examples involving revoked honors.

**Results:**

Three distinct state-facilitated growth pathways were delineated: Ladder-shaped, Folded-ladder, and Inverted-ladder. An interaction-driven model predominated (64.76%), highlighting a core mechanism of “strategic alignment” between individual agency and state-facilitated opportunity structures. A significant geographical imbalance was identified, with awardee distribution showing a moderate positive correlation with provincial population but pronounced over-representation of major municipalities. The celebrated virtues, such as Dedication and Patriotism, represent a fusion of universal PYD constructs with collectivist values.

**Conclusions:**

This study foregrounds the institutional construction of exemplary youth. It contributes to a more culturally contextualized and theoretically robust understanding of PYD. The findings offer evidence-based insights for youth policy and the dynamic governance of state recognition systems, while critically engaging with the sustainability of state-sponsored exemplarity.

## 1 Introduction

This study investigates positive youth development (PYD) in China by analyzing exemplary recipients of the China Youth May Fourth Medal. As a major paradigm shift in developmental science since the 1990’s, PYD moves beyond a deficit perspective to focus on youth strengths, competencies, and potential (Lerner et al., 2011). Grounded in relational developmental systems theory, PYD views development as a dynamic process of interaction between individuals and their contexts (Overton, 2013), operationalized through core frameworks like the “Five Cs” (Competence, Confidence, Connection, Character, Caring) and the Developmental Assets model (Lerner et al., 2005; Benson et al., 2011). While PYD research has expanded globally, studies within China remain limited and predominantly variable-centered, often overlooking individual heterogeneity and life-course perspectives. Furthermore, prevailing PYD models, largely developed in Western individualistic contexts, require critical examination and adaptation within collectivist societies like China, where development is deeply embedded in national goals and collective values (Shek et al., 2023).

To address these gaps, this study focuses on the 1,219 recipients ((1997–2023)) of China’s highest youth honor, the May Fourth Medal. This cohort, including senior leaders, leading scientists, and prominent business leaders, constitutes the officially recognized and celebrated exemplars of Chinese youth, representing the state-endorsed archetype of positive development. Through a biographical analysis of these exemplary individuals, this research aims to: (1) elucidate the characteristic profile of this state-recognized elite; (2) identify their key growth trajectories; and (3) explore the interplay of personal qualities and environmental factors in their development. It deconstructs this nationally endorsed model to advance a culturally contextualized understanding of PYD.

## 2 Literature review and theoretical framework

### 2.1 Positive youth development theory: a review of its origins, core models, and key research

The study of youth development underwent a fundamental paradigm shift in the late 20th century. Moving away from a dominant deficit-oriented approach that focused on preventing problems like delinquency and maladjustment, the field embraced positive youth development (PYD) (Lerner et al., 2011). PYD, influenced by positive psychology and relational developmental systems theory, reframed the core question from “how to fix problems” to “how to cultivate strengths” (Lerner et al., 2003). It conceptualizes youth as active agents with plasticity and potential, whose thriving stems from the dynamic interaction between individual assets and supportive environments. This holistic, strength-based paradigm does not ignore risk but posits that building positive attributes forms a robust foundation for long-term wellbeing and social contribution (Roth and Brooks-Gunn, 2003), setting the stage for operational models of thriving.

Positive youth development is operationalized through complementary models that specify the qualities and conditions for thriving. The Five Cs model (Competence, Confidence, Connection, Character, Caring) provides a multidimensional framework for analyzing the personal qualities of exemplary youth (Lerner et al., 2005). Competence and Confidence relate to effective functioning and self-efficacy; Connection and Caring to social embeddedness and prosocial orientation; and Character to internalized values and integrity. This model is particularly useful for deciphering the celebrated moral and spiritual qualities of role models. However, its primary focus on individual attributes underscores the need to integrate it with frameworks that more explicitly address contextual influences (Damon, 2004).

The Developmental Assets framework fulfills this need by emphasizing the ecological conditions that foster PYD (Benson et al., 2011). It distinguishes between external assets (support, empowerment, boundaries, opportunities provided by families, schools, and communities) and internal assets (commitment to learning, positive values, social competencies cultivated by the individual). This framework is invaluable for analyzing how multi-level contexts—from family upbringing to national policies—shape developmental trajectories. It also highlights the socio-legal dimensions of development, as laws, policies, and institutional norms constitute critical “external assets” that structure opportunities, define valued contributions, and legitimize pathways to recognition. A socio-legal inquiry thus complements this framework by examining how the state, through its legal and policy apparatus, actively constructs the “opportunity structures” and “normative expectations” that are central to the asset-building process.

A critical frontier in PYD research is its cross-cultural applicability. Core models like the Five Cs, developed in individualistic Western contexts, may manifest differently in collectivist societies like China (Scales et al., 2017). For instance, Connection may emphasize duty and alignment with collective missions over peer affiliation, and Confidence may be rooted in a sense of responsibility rather than personal assertiveness. Cross-national studies confirm that while the structural dimensions of PYD are identifiable globally, their configuration and salience are culturally patterned (Buenconsejo et al., 2025). Therefore, applying PYD to analyze Chinese exemplary youth, such as “May Fourth Medal” recipients, serves as a crucial test and refinement of the theory itself.

This engagement necessitates a socio-legal extension of the PYD lens. In China, the state plays a constitutive role in defining and facilitating positive development through top-down legal frameworks, ideological campaigns, and formal recognition systems like national awards. The “May Fourth Medal” is not merely a neutral honor but a socio-legal institution that performs key functions: it operationalizes national strategies by rewarding aligned behaviors, legitimizes state priorities, and scripts normative life courses for youth. From this perspective, the celebrated “Five Cs” and “Developmental Assets” are not just naturally occurring traits and supports but are often cultivated within, measured against, and amplified by a specific socio-legal order. Thus, studying Chinese exemplary youth through an integrated PYD and socio-legal lens does more than apply a Western theory; it enriches PYD by incorporating the formative power of state-led normative and legal structures, advancing toward a more globally inclusive and institutionally attuned understanding of how youth thrive.

### 2.2 Youth development in the Chinese context: policy, culture, and social transformation

The youth is frequently mentioned by the public and has been of concern throughout the Chinese Communist Party since 1980, notably after the cultural revolution (Liu, 1984). According to the seventh national census in 2020, China’s population of youth aged 14–35 was about 400 million, making up 28.4 percent of the total population. The development of Chinese youth cannot be fully understood without considering the unique interplay of state-led policy frameworks, cultural traditions, and rapid social transformations. This context creates a distinctive environment for PYD that differs in significant ways from Western models. This section rooted in the Communist Party of China “Governing Youth principle,” along with cultural values like “family-nation sentiment” and “collectivism,” and the macro-social narrative of “national rejuvenation,” collectively shape the pathways, goals, and manifestations of positive development for Chinese youth.

First, within the framework of the state-led youth policy, the most direct factor shaping youth development in China is the comprehensive policy system established under the principle of the Communist Party of China Governing Youth, which differs from Western PYD models that often place emphasis on individual autonomy and community-led support. As evident in the shift from the Well-off Society narrative (1979–2011) to the China Dream narrative (2012–present), youth Development objectives have transitioned from “all-around development” to a focused emphasis on “innovation capacity building” (Li, 2020). As the first national-level youth development specialized plan, Medium-and-Long-term Youth Development Plan (2016–2025) institutionalizes the concept of “Youth Priority Development”. This represents a state-led, proactive investment in youth as a strategic resource for national development. For instance, during the “13th 5-Year Plan” and “14th 5-Year Plan” periods, several provincial, municipal, and county-level plans for the first time included dedicated sections or chapters on youth development, guided by the vision of “making cities more friendly to youth, and youth more capable in cities,” aimed at building a Youth Development City model, also triggered a “talent recruitment war” among cities (Liu and Jin, 2019).

Second, cultural traditions infuse with a collective-oriented value system, shaping how youth development is framed and practiced. Individualistic and collectivistic cultures are broadly characterised by the priority of individual over group interests, versus the priority of ingroup interests over the interests of the individual (Hofstede, 1991; Triandis, 1995). Chinese collectivist culture was heavily influenced by Confucian moral values and principles (Chan et al., 2022). In the schoolrooms, the hallmarks of collectivist education were everywhere: uniforms, strict timetables, long hours of study and the unrelenting push toward exams. The highest aspiration for a young person, as promoted in official discourse is to integrate personal dreams into the great dream of the country and the nation. Such as the spirit of creation, the spirit of struggle, the spirit of unity, and the spirit of the Chinese dream, stress on the importance of national rejuvenation and of the social responsibility of individuals, where personal aspirations are often expected to align with and contribute to national goals and collective wellbeing. In the realm of youth development, this collectivist orientation manifests as an emphasis on integrating the small self into the larger self of the nation and the people. The state actively fosters this integration by creating sanctioned “battlefields” for youth to contribute, such as initiatives in rural revitalization, technological innovation, and pandemic prevention, where individual struggle is given higher meaning and social recognition when directed toward national priorities. Consequently, positive development is measured not only by individual thriving (e.g., competence, confidence) but also by the youth’s demonstrable contribution to collective goals. This is a distinctive feature of the Chinese PYD model.

Third, rapid social transformations, transitioning from a labor-intensive to a technology-intensive manufacturing economy, driven by innovation rather than being reliant on imported materials in china (Han and Deng, 2025), have introduced unique demands and opportunities for PYD. These transformations require young people to possess not only advanced technical skills but also the ability to adapt to rapidly changing industrial landscapes, fostering a need for PYD programs that emphasize innovation, lifelong learning, and cross-disciplinary collaboration. In terms of emphasizing innovation, China has recently emerged as a key power that drives R&D and innovation in the world (Li et al., 2020), and youth are an important driving force in this process. The General Office of the Communist Party of China Central Committee and the General Office of the State Council released Several Measures on Further Strengthening the Cultivation and Utilization of Youth Science and Technology Talents in August 2023, encourage young scientific and technological talents to actively engage in the practical processes of economic and social development, identify key scientific challenges based on real-world needs, conduct original research, achieve technological breakthroughs, promote the transformation of scientific achievements, and apply their academic work to the development of the nation. (General Office of the Communist Party of China (CPC) Central Committee, and General Office of the State Council of the People’s Republic of Chinaand, 2023). Policies such as opening science and technology museums to youth and integrating digital skills training into youth work are tailored to address the needs of a digitalized, innovation-driven economy. Additionally, China’s urban-rural divide has shaped youth development to include targeted efforts, such as community volunteer services and volunteer service in western China, policies leave autonomy to society by applying more symbolic and incentive tools (Zhang et al., 2024).

In summary, this context fosters a distinct model where the ultimate manifestation of “positive development” is the cultivation of youth who are well-rounded and morally grounded, who shoulder heavy responsibilities and prove themselves capable, and who possess a global vision and a sense of duty to the world. These characteristics delineate a framework that cannot be fully captured by Western PYD models alone, as it intentionally prioritizes aligning personal aspirations with national goals, fostering a collective identity, and channeling youthful vigor toward addressing the specific challenges and opportunities of a rapidly modernizing great nation.

### 2.3 Research on youth role models and the research framework of the article

Research on the interplay between advanced role models and youth development has consistently identified three core mechanisms through which role models exert transformative influence on young people, each supported by robust theoretical frameworks and empirical findings. Firstly, the demonstration effect, where the commendable actions of role models offer young people specific behavioral templates. Rooted in Bandura’s (1977) Social Learning Theory, this mechanism posits that young people acquire new behaviors not merely through direct experience, but by observing and imitating the observable, commendable actions of role models—actions that serve as concrete “behavioral templates”. Secondly, the goal inspiration effect, where the outstanding achievements and spiritual qualities of role models stimulate young people’s intrinsic motivation to strive for excellence. And the spiritual qualities embodied by role models—such as resilience in the face of setbacks, dedication to long-term goals, and humility amid success—reinforce the perceived attainability of these benchmarks (Schunk, 1987). Thirdly, the value identity effect, where the values embodied by role models resonate with and are internalized by young people, thereby shaping their own value systems. Hitlin and Piliavin (2004) argued that value identification occurs when role models’ expressed values (e.g., social responsibility, equity, or a commitment to lifelong learning) align with young people’s latent value needs, triggering a sense of “moral congruence.” Notably, these three mechanisms do not operate in isolation but interact synergistically to amplify role models’ influence. The potency of these mechanisms is vividly demonstrated in the study of international elite groups, where the recognition and visibility of role models profoundly shape collective aspirations and fields of endeavor.

For example, studying the visibility of Nobel laureates in the sciences is an extraordinary gateway for understanding the transformations in the public image of science – and of scientists – throughout the 20th century and also understanding how the Prize itself contributed to shape such image (Bucchi, 2018). Decades-long reiterated attention to the prizes and the laureates in the public press, whether the recipient was Albert Einstein or a countryside doctor toiling for years in obscurity, whether the prize was worthily given or awarded to work later disproved, or whether we even remember the honorees today, the Nobel defined the image of science in the twentieth century, an image that still lives in all sorts of fascinating ways today. Zuckerman (1996/1977) tracks the careers of all American laureates who won prizes from 1907 to 1972, examining the complex interplay of merit and privilege at each stage of their scientific lives and the creation of the ultra-elite in science. Rahkamo (2016) using grounded theory, identified the following factors as key contributors to the development of Olympic champions: (1) reflective inquiry and cognitive engagement with ideas, (2) insight, (3) systematic application of skills, (4) self-confidence, (5) intrinsic motivation, and (6) sustained effort.

While the interplay of role model mechanisms is observable globally, its manifestation is uniquely shaped by specific socio-political and cultural contexts. In China, the institutionalized use of advanced role models represents a particularly powerful and deliberate application of these psychological and social principles. The use of advanced role models, such as labor models and medal recipients, has a long and profound tradition in China’s youth education and development work. It serves as a crucial means for ideological and political education, value guidance, and spiritual inspiration. This approach is deeply rooted in China’s political and cultural context. Historically, from model workers like Lei Feng in the 1960s to “May Fourth Medal” recipients and “Upward and Virtuous Good Youth” in the new era, the selection, promotion, and learning from advanced role models have consistently been an important tool for the Communist Youth League and the Communist Party of China to guide, unite, and inspire young people. The core value of this approach lies in making abstract core socialist values and the spirit of the times concrete, visible, and relatable. By showcasing the exemplary deeds of real individuals, it provides young people with tangible benchmarks for emulation, helping them “fasten the first button of life” correctly. The communication methods also leverage new media, using youth-friendly language and methods to tell the stories of role models well, making this traditional method of ideological education radiate with new vitality in the new era. Within this structured Chinese context, the three core mechanisms—demonstration, goal inspiration, and value identity—are strategically activated through state-supported recognition systems. Therefore, studying the “May Fourth Medal” recipients, as the highest-level advanced youth role models in China, is not only a continuation of this tradition but also an ideal window to observe the characteristics and pathways of PYD within the specific political and cultural context of China.

Based on the comprehensive literature review, this study constructs an integrated analytical framework to guide the empirical examination of the 1,219 China Youth May Fourth Medal recipients, adopting a socio-legal inquiry perspective—building on the mechanisms of role model influence—to examine the Medal not merely as an honor, but as a core component of China’s normative ordering for youth development. This framework is predicated on the dialectical integration of core concepts from Western PYD theory with the distinctive elements of the Chinese youth development context. Posits that the Medal recipients embody the successful outcomes of this synergistic interaction. Their development is not merely a manifestation of universal PYD principles but is profoundly shaped by the specific goals, values, and opportunity structures within China. Accordingly, the framework is organized around three interconnected analytical dimensions, as summarized in the Table 1, which will structure the subsequent biographical analysis.

**TABLE 1:** An integrated analytical framework for examining positive youth development (PYD) in the Chinese context.

Analytical dimension	Theoretical anchorage	Core research focus
1. Image characteristics	PYD (5Cs): to assess universal traits of thriving youth. Chinese context: to examine how national priorities for balanced development shape the composite image of recognized elites.	What are the demographic and qualitative characteristics of the awardees? How does this profile reflect the interplay between individual competence (PYD) and state-led goals for an equitable and comprehensive model youth?
2. Growth trajectory	PYD (Developmental Systems Theory): to understand development as a dynamic person-context interaction. Chinese context: to analyze how national strategies create sanctioned pathways and critical turning points.	What are the typical career pathways? How do individual agency and national policy-driven opportunities interact to shape these distinct trajectories towards elite status?
3. Spirituality and values	PYD (Character, Caring/Contribution): to explore positive values and prosocial orientation. Chinese context: to investigate the internalization of collectivist values and official ethos as the ultimate expression of positive development.	What core spiritual qualities are celebrated in the awardees’ deeds? How do these qualities represent a fusion of universal PYD virtues with the specific expectations of “contributing to the national rejuvenation” in China?

This framework serves as a guiding lens for the empirical analysis. It allows us to systematically investigate how the image characteristics of the awardees represent a selection outcome influenced by both PYD criteria and Chinese policy goals; how their growth trajectories are forged through the interplay of personal initiative and nationally-created opportunities; and how their spiritual qualities embody the fusion of universal PYD virtues with distinctive Chinese cultural and ideological expectations. Ultimately, employing this framework will enable a critical discussion on how the Chinese case can test, refine, and potentially enrich the broader, predominantly Western-derived, PYD theory by introducing a culturally and politically contextualized perspective.

## 3 Data and methods

### 3.1 Research design

This study employs a mixed-methods content analysis approach to systematically examine media reports on 1,219 recipients of the China Youth May Fourth Medal. Grounded in curriculum vitae (CV) analysis, which provides a rich source of longitudinal information addressing nearly all dimensions of a researcher’s career (Bozeman et al., 2001), and proposes a conceptualization of the life course as a set of behavioral processes characterized by interdependencies that cross time, life domains, and levels of analysis (Bernardi et al., 2019). The research aims to quantitatively profile the demographic characteristics of awardees while qualitatively interpreting their developmental trajectories and core spiritual qualities. This design enables a comprehensive exploration of how exemplary youth in China are shaped by individual, social, and institutional factors, addressing the central question: What makes outstanding youth outstanding?

### 3.2 Data sources and sample

The analysis is based on a comprehensive dataset of China Youth May Fourth Medal recipients, covering the first 27 award cycles from 1997 to 2023 (*N* = 1,219)

Data were collected from official and authoritative sources, including: people.cn, China Youth Online, and CPC News; the China Youth Daily and other state-sanitized media archives; official announcements from the Central Committee of the Communist Youth League and the All-China Youth Federation.

Collected data included: basic demographic details: gender, age at award, ethnicity, political affiliation, educational background, geographic region, and industry sector. Thus, image is a multidimensional construct generated from a wide range of how an image object’s attributes are perceived (Dowling, 1986; Kennedy, 1977).

Narrative materials: detailed descriptions of recipients’ achievements, career paths, and post-award developments as reported in media profiles. In the context of media profiles documenting recipients’ achievements, career trajectories, and post-award progress, news media organizations play a pivotal role as both recorders and interpreters—their ability to fulfill this role is shaped by how audiences perceive their image, particularly along dimensions of credibility, usefulness, and empathy (Shin, 2022).

### 3.3 Variable measurement and operationalization

The operational definitions of all variables are provided in Table 2 to ensure systematic coding and analysis.

**TABLE 2:** The variable operation diagram of positive youth development (PYD).

Variable category	Variable name	Operationalization
Image characteristics	Gender	Recorded as male or female.
	Age at award	Calculated based on award year and birth year.
	Geographic distribution	Categorized by province with additional analysis using the Eastern, Central, Western classification.
	Educational background	Classified into seven levels: primary school or below, junior high, senior high/vocational, associate degree, bachelor’s, master’s, and doctoral degree.
	Industry sector	Coded into 12 categories based on the *Industrial Classification of the National Economy* (2002), e.g., public administration, industrial services, agriculture, tech innovation, etc.
Growth trajectory	Trajectory type	Qualitatively coded into three types based on key life-course transitions: 1. Ladder-type: steady advancement within a single field. 2. Folded-ladder-type: cross-sector or multi-stage career shifts. 3. Inverted-ladder-type: overcoming adversity or making late-stage breakthroughs.
Spiritual qualities	Core values	Coded based on descriptors in media profiles, aligned with the *New Era Civic Morality Construction Guideline* (2019). Categories included: patriotism and dedication, professional devotion, innovation, perseverance, self-reliance, public service, and familial devotion.
Growth factors	Influencing factors	1. Internal-driven: emphasizing personal effort and resilience. This category was operationalized for cases where growth was primarily attributed to sustained individual initiative, self-improvement, and perseverance, with external conditions playing a facilitative rather than deterministic role. 2. External-driven: highlighting the dominant role of external opportunities. This category refers to instances where an individual’s trajectory appears overwhelmingly shaped by structural factors, privileged access to resources, or socio-familial platforms, with personal agency being less pronounced in narrative accounts. 3. Interaction-driven: highlighting combined effects of individual agency and external support. This category was applied when growth narratives demonstrated a salient interplay between personal striving and enabling external conditions (e.g., family background, policy support, or critical organizational platforms).

### 3.4 Analytical methods

The operational definitions of all variables are provided in Table 2 to ensure systematic coding and analysis.

Both quantitative and qualitative techniques were applied:

Descriptive statistics: Frequencies, percentages, and means were calculated for demographic variables using SPSS 28.0 to outline the cohort’s profile.

Inferential statistics: Chi-square tests were used to examine associations between categorical variables (e.g., industry and spiritual qualities). Binary logistic regression analyzed the effects of time, education, age, region, industry, and trajectory type on growth factors.

Qualitative analysis: Inductive coding and typological analysis were applied to narrative data to identify patterns in growth trajectories and value expressions. Case illustrations were used to contextualize quantitative trends.

This mixed-methods approach allows for both macro-level trend identification and micro-level insight into the mechanisms underlying positive youth development in China.

## 4 Findings

### 4.1 Demographic profile and image characteristics of awardees

To delineate the composite profile of China’s exemplary youth, this section applies the first dimension of our integrated framework (see Table 1), which combines the PYD lens with the Chinese context that emphasizes national priorities for balanced development. Our analysis of demographic and career data seeks to reveal how the recognized image of elite youth is shaped by the interplay between individual competence and state-led goals for an equitable and comprehensive model.

Analysis of the 1,219 award recipients reveals a composite profile of China’s recognized exemplary youth. In terms of gender distribution, male awardees constitute a significant majority (73.42%, *n* = 895), while female awardees represent 26.58% (*n* = 324). Gehmlich and Krause (2024) statistics gender distribution of 8,747 awardees of 345 scientific awards, medals and prizes between 1,731 and 2,020, still far from gender parity amongst the recipients of these awards, and only 15.4% of the recipients of the considered scientific awards and prizes were women. However, a longitudinal examination indicates a clear trend towards gender parity over the 27-year period, with the male-to-female ratio showing a progressive narrowing, from a historical peak of 9:1 to the overall ratio of 2.76:1 among all awardees. However, a longitudinal examination indicates a clear trend toward gender parity over the 27-year period, with the male-to-female ratio showing a progressive narrowing. This evolving pattern towards gender parity aligns with and can be partly attributed to a series of national policies aimed at promoting women’s participation in all spheres of society. For instance, the landmark Law on the Protection of Rights and Interests of Women was enacted in 1992, serving as a foundational statute, and has been revised three times in 2005, 2018, and 2022. Since 1995, the Chinese government has instituted a series of dedicated national programs for women’s development, systematically working toward gender equality in political, economic, cultural, social, and family life.

Regarding age structure, the average age at the time of award receipt is 32.99 years. A notable divergence exists across sectors. For instance, recipients in “Sports” exhibit the lowest average age (25.07 years), reflecting the youthful peak of athletic careers. Which is consistent with the findings of a study on the age of peak performance in Olympic sports, a study on 2012 Summer Olympics athletes showed that 72% of Olympic athletes aged between 20 and 30 years (the main stage of youthful peak), and 99% aged below 40 years (Longo et al., 2016). While those in “Science, Technology, and Energy” display the highest average age (36.32 years), indicative of the prolonged accumulation period required for scientific breakthroughs, this phenomenon can be explained by the cumulative advantage theory in scientific productivity research. The theory suggests that researchers who receive professional recognition (such as project funding, international collaboration opportunities) tend to maintain higher productivity as they age, and their ability to achieve major breakthroughs improves with the accumulation of resources and experience. A study on Norwegian academics confirmed that full professors maintain the highest productivity in middle age far exceeding that of younger researchers (Kyvik, 1990). This is further supported by Nobel Laureate data: the average age of 178 Laureates (across physics, chemistry, medicine, economics) when conducting prize-winning research was 44.1 ± 9.7 years, a middle-age stage where they had accumulated sufficient professional capital (Bjørk, 2019). Statistical analysis confirms a significant correlation between industry and age distribution (*r* = 0.350, *P* < 0.001).

**FIGURE 1:**
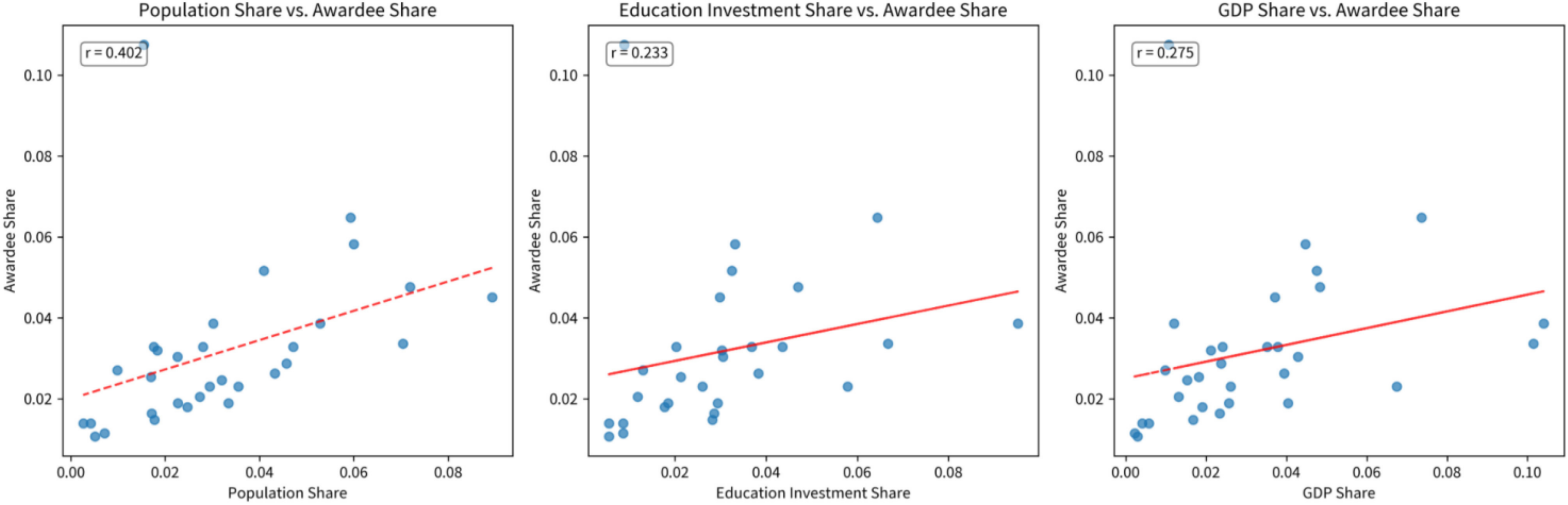
Correlation analysis between awardee share and key provincial indicators across Chinese provinces.

Geographically, awardees are distributed across all provincial-level administrative regions, yet with marked disparities. This pattern, a product of socio-legal design, is shaped by the official selection mechanism—operating through provincial-level nominations followed by national evaluation—and embeds a tension between meritocratic principles and redistributive logic. In theory, this could lead to a distribution proportional to provincial population bases. However, our findings reveal a more complex picture: major population provinces like Guangdong, Shandong, and Henan are not the top contributors, whereas municipalities like Beijing, Shanghai, and Tianjin, with smaller populations, contribute a disproportionately high number of awardees. Statistical correlation analysis based on 2025 provincial data provides quantifiable support for this observed imbalance. As shown in Figure 1, awardee share across provinces demonstrates a modest positive correlation with 2025 population share (*r* = 0.402). The correlations with 2025 provincial GDP share (*r* = 0.275) and 2025 education investment share (*r* = 0.233) are also positive but weaker. This pattern indicates that while demographic scale provides a foundational correlate, the distribution of awardees is not solely determined by population size. Notable exceptions persist, particularly the over-representation of major municipalities, suggesting that other socio-political and institutional factors significantly shape the geographical outcomes of the national recognition system. This over-representation aligns with its status as a national capital and a hub for talent, policy, and media resources, and is a stark manifestation of the “Matthew effect” in China’s talent distribution. Furthermore, the selection process appears to incorporate a compensatory logic towards remote regions. For example, Tibet, Qinghai, Hainan, and Ningxia, which together account for only 1.90% of China’s population, contribute 5.00% of the awardees, highlighting a deliberate tilt in the selection process. The distribution also shows a significant correlation with industry (*r* = 0.260, *P* < 0.001), a key finding of our analysis. This correlation reflects China’s regional economic structures. Developed provinces with larger tertiary sectors, such as Jiangsu and Zhejiang, have more recipients in categories like “Commercial Innovation.” Conversely, major agricultural provinces like Henan dominate the “Agriculture and Rural” category. This pattern aligns with the structural disparities in provincial economies across China (Sakamoto, 2010). The geographical concentration of talent and resources in coastal and urban hubs (Nam, 2017), coupled with the negative spillover effects on surrounding areas (Shang and Liu, 2024), further contextualizes the underlying spatial inequalities in resources essential for PYD, such as education and opportunity.

Among middle-income countries China is the one that perhaps has carried out the largest investment in HE, and expanded the most, in recent times (Huang et al., 2022). But in terms of educational background, the “highly educated” image is not absolute, there are likely to be many youth for whom participating in organized activities has a very large and positive effect (Mahoney et al., 2006). Bachelor’s degree holders form the largest group (37.93%), followed by postgraduates (27.92%), while associate degree holders account for 15.39%. However, the proportion of associate degree holders among award winners across all previous sessions also shows a fluctuating downward trend. A significant correlation exists between industry and educational attainment (*r* = 0.265, *P* < 0.001). Sectors like “Education and Culture” and “Scientific Research” feature the highest proportions of doctoral degree holders (47.92%, 35.63%, respectively), whereas “Industrial Services” and “Sports” have significant representation of individuals with senior high school education or below (38.27%, 44.39%, respectively), demonstrating that exemplary contributions are valued across a spectrum of knowledge structures.

### 4.2 Typology of growth trajectories

Guided by the second dimension of our analytical framework (Table 1), which centers on the growth trajectory, this section examines career pathways through the theoretical lens of developmental systems theory. This perspective conceptualizes development as a dynamic person-context interaction.

Based on the actual circumstances of the research subjects, particularly the significant “turning points” identified in the growth journeys of the “May Fourth Medal” recipients, this study conceptualizes personal growth through four key developmental nodes: (1) the Starting Point (background information such as family and education), (2) the Growth Period (experiential information from work and life), (3) the Award Moment (the landmark event of receiving the Medal), and (4) Post-Award Development (sustained growth following the recognition). The operationalization and classification of growth trajectories proceeded through the following steps: First, narrative materials for each awardee were systematically coded according to these four nodes to extract key events, turning points, and sustained endeavors. Second, through cross-case comparative analysis, distinct patterns were identified based on the dynamics of growth momentum, the nature of critical transitions, and the overall morphology of the life-course narrative. The classification into three prototypical trajectories was derived inductively from these patterns: the Ladder-shaped trajectory was assigned to cases demonstrating steady, cumulative progression across the nodes; the Folded-ladder trajectory to cases characterized by prolonged dedication in a specific domain culminating in a transformative leap triggered by a critical event [often at Node (3)]; and the Inverted-ladder trajectory to cases where a significant early setback or adversity [at Node (1) or (2)] was overcome, leading to a decisive reversal and breakthrough, as exemplified by awardee An Fengzhen (12th), who overcame a disabling traffic accident during her growth period to later achieve breakthrough athletic success and post-retirement contributions. This method ensures that the typology is grounded in observable, coded biographical data rather than a priori assumptions.

**FIGURE 2:**
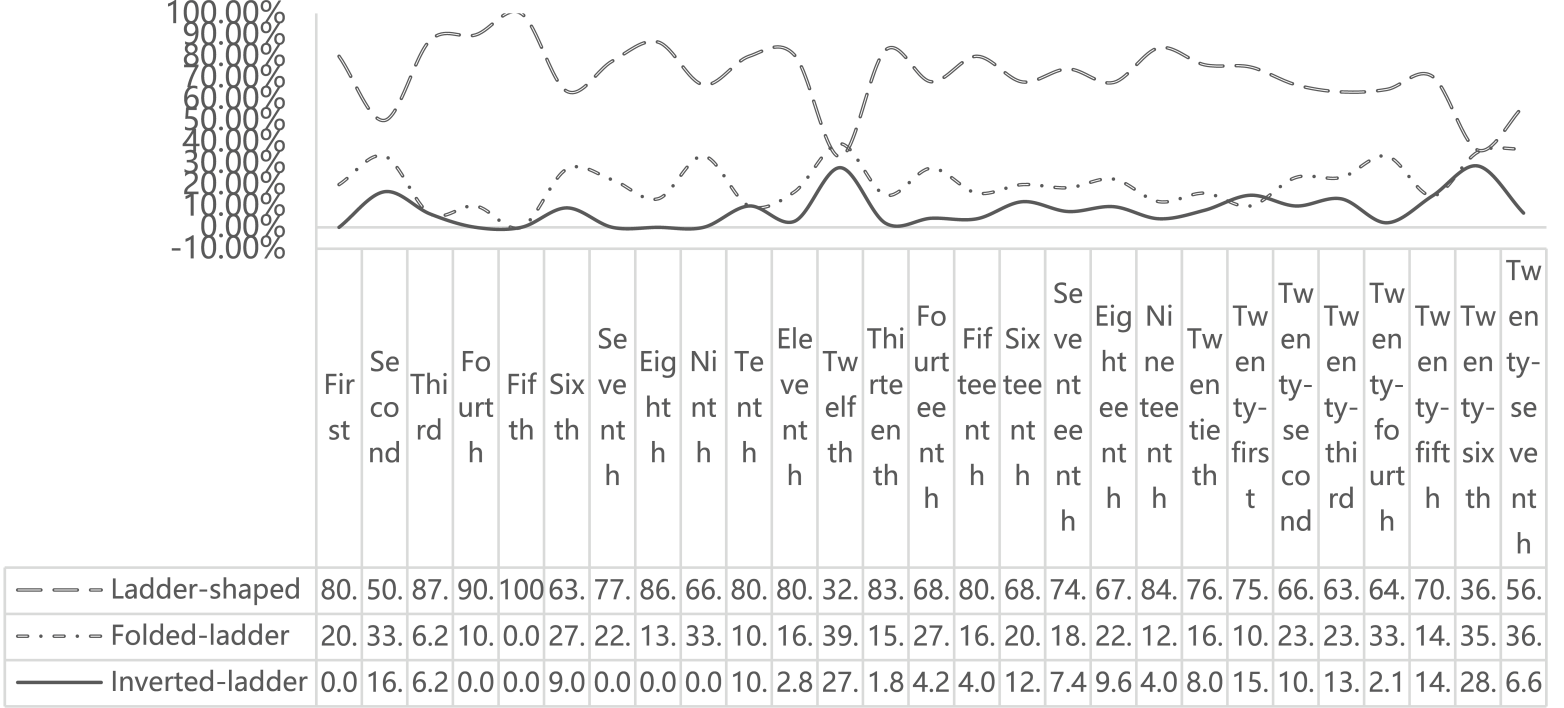
Growth trajectory types of 1,219 China Youth May Fourth Medal recipients.

A qualitative analysis of the progression through these nodes revealed three distinct prototypical growth trajectories (in Figure 2), each closely intertwined with the core constructs of implicit theory beliefs, possible selves, and action orientation identified in the study by Bhattacharya and Mehrotra (2013). These trajectories—identified as the Ladder-shaped, characterized by steady, progressive advancement within a specific field; the Folded-ladder, marked by significant cross-sector or functional transitions; and the Inverted-ladder, defined by overcoming major adversity to achieve breakthroughs—reflect how individuals’ perceptions of self-changeability, images of their future selves, and proactive efforts concretely shape their intentional personal growth processes within a Chinese context.

(1) Ladder-shaped trajectory (61.11%): Characterized by steady, progressive advancement within a specific field or system. This trajectory emphasizes gradual accumulation of expertise and seniority. Individuals following this path typically endorse the view that personal attributes (e.g., professional skills, emotional regulation abilities) are malleable and can be developed through sustained effort. From the perspective of PYD theory, this trajectory vividly reflects the synergistic enhancement of internal and external developmental assets. The progressive accumulation of professional competence and the strengthening of connections within a specific field or system align with the core concepts of the 5Cs model (Competence, Confidence, Connection, Character, and Caring), demonstrating how a structured environment and personal effort jointly promote the comprehensive development of youth. Furthermore, this progressive path underscores the great importance of long-term, sustained investment and the construction of a supportive environment in the cultivation of outstanding youth within a specific Chinese context. Wang et al. (2025) pointed out that Chinese youth in the new era have strong confidence in future development and optimistic expectations for their personal status.
(2) Folded-ladder trajectory (26.09%): This trajectory is marked by significant cross-sector or functional transitions, demonstrating remarkable adaptability and the integration of diverse experiences into a unique career composite. A distinctive feature of this path, as exemplified by awardees from typical occupational groups such as workers, farmers, drivers, nurses, and soldiers, is the dynamic interplay between steadfast dedication in ordinary times and heroic action in critical moments. These individuals exemplify the ability to “remain grounded during mundane times”—showcasing years of unwavering commitment, rootedness in their posts, and dedicated service or care in their daily roles. Simultaneously, they demonstrate the capacity to “rise to the occasion when it matters most”—embodying a sense of mission that drives them to charge ahead and assume responsibility during pivotal events. From a PYD perspective, this trajectory vividly illustrates the dynamic development of adaptability and resilience. The ability to navigate significant transitions and integrate diverse experiences underscores a high level of Competence in managing change. Furthermore, the juxtaposition of long-term perseverance with proactive leadership in crises reflects a strong Character and a deep sense of Connection to the well-being of their communities and values. This pattern demonstrates that positive development is not linear but can involve synthesizing seemingly disparate life experiences into a coherent and purposeful whole, ultimately leading to profound Contribution. The Folded-ladder trajectory, characterized by the synergy of steadfastness and adaptability, underscores the importance of volitional processes in Chinese youth development. Our findings resonate with research highlighting the role of belief in free will in fostering perseverance among Chinese adolescents (Li et al., 2018). This suggests that the celebrated “Character” and “Connection” in the Chinese context may be deeply intertwined with culturally embedded cognitive schemas that emphasize individual agency within a collective framework, thus enriching the Western-derived PYD model.
(3) Inverted-ladder trajectory (12.80%): Defined by overcoming significant adversity or achieving major breakthroughs later in the career, often following early setbacks, the Inverted-ladder Trajectory exemplifies the phenomenon of post traumatic growth (PTG)—the idea that trauma and adversity can engender profound psychological development. This concept, which gained empirical traction in the late 20th century and was operationalized by Tedeschi and Calhoun’s (1996) Posttraumatic Growth Inventory (PTGI), helps to frame the transformative growth observed in this path. The PTGI measures growth across five domains: spiritual development, increased intimacy and connection with others, enhanced personal strength, appreciation of new possibilities, and a renewed appreciation of life. These dimensions map powerfully onto the trajectory of these individuals. Individuals may initially hold entity theory beliefs after early failures, perceiving their feared possible selves as vivid and unavoidable. However, critical turning points prompt a shift toward incremental beliefs: they begin to view setbacks as “opportunities to build skills,” thereby developing an enhanced sense of personal strength and discovering new possibilities for their lives—core aspects of PTG. Their journey underscores the self-regulatory role of possible selves: as they progress, their desired selves become more salient, and they actively link current actions to long-term transformation, often leading to increased intimacy and connection with others and a renewed sense of priorities. In essence, the Inverted-ladder Trajectory offers a powerful testament to the core PYD principle that adversity, when processed through a supportive framework, can become a potent catalyst for developing resilience, empathy, and purpose. This aligns with the Chinese cultural emphasis on resilience and overcoming adversity, demonstrating how the universal mechanisms of PTG and PYD are expressed within a specific socio-cultural context. The trajectory thus embodies a culturally-inflected model of growth where profound contribution arises from the successful navigation of challenge.

These three trajectory archetypes emerged systematically from narrative data through rigorous analysis, rather than being predetermined. Their distinct profiles and alignment with established psychological constructs confirm that this typology is data-driven and empirically grounded, rather than a simple subjective classification. These findings uncover culturally distinct patterns of youth achievement and provide a methodologically sound basis for understanding how individual agency, contextual support, and life experiences collectively shape positive developmental outcomes.

### 4.3 Core spiritual qualities and contributing factors

This section addresses the third dimension of our framework (Table 1), focusing on the spirituality and values embodied by the awardees. It employs the PYD constructs of Character and Contribution to identify universal virtues, while simultaneously investigating how these virtues are infused with and expressed through collectivist values and the official ethos in China.

Content analysis of award citations identifies a set of core spiritual qualities celebrated in these role models. The most prominent are Dedication and Professionalism (28.7%), Pioneering and Innovation (21.9%), and Patriotism and Contribution (21.8%). Other qualities include Self-reliance (12.6%), Hard Work and Perseverance (11.8%), Public Service (2.7%), and Filial Piety and Family Love (0.7%). Building on this, further research reveals that the core spiritual qualities of 1219 award winners are independent of award sessions and regions (*P* = 0.173 and 0.043, respectively), yet show significant correlations with gender, educational background, age, and industry (all *P* < 0.001).

(1) In terms of gender, male winners demonstrate more convergent Dedication and Professionalism as well as Pioneering and Innovation. In contrast, female winners exhibit greater convergence in Public Service and Filial Piety and Family Love. This pattern reflects socially and culturally constructed gender role expectations (Barbee et al., 1993), whereby male achievement narratives are framed around mastery and agency in the public sphere, and female exemplarity is inherently tied to the expression of relational and caring virtues across private and public domains.
(2) When comparing educational backgrounds, on one hand, individuals with lower education levels such as primary and junior high school degrees gain social recognition through Self-reliance (49.0%). On the other hand, those with higher education like master’s and doctoral degrees achieve life values through Patriotism and Contribution (42.7%). This divergence illustrates two distinct pathways to recognition: individuals with lower formal education often gain status through the moral capital of “Self-reliance,” a compensatory non-material asset that offsets educational disadvantages in social stratification (Coleman et al., 1966), while those with higher education are typically channeled to convert specialized knowledge into national service.
(3) Regarding age distribution, the average age varies across qualities: Pioneering and Innovation (35.63), Hard Work and Perseverance (34.16), Dedication and Professionalism (33.89), Public Service (33.44), Self-reliance (27.16), and Filial Piety and Family Love (26.5). This gradient reflects a lifecourse logic in which qualities associated with personal agency and private virtues are recognized earlier, while those requiring accumulated expertise and systemic embeddedness emerge in more established career stages.
(4) From the perspective of industry differences, Patriotism and Contribution are concentrated in Sports and Athletics (37.4%) and Science, Technology and Energy (29.3%); Dedication and Professionalism are concentrated in Administrative and Public Services (35.4%) and Military and National Defense (25.2%); and Filial Piety and Family Love are more clustered in Education and Culture (87.5%), indicating that different sectors are entrusted with distinct social functions and national expectations.

Analysis of contributing factors indicates that the vast majority of cases (64.76%) are best explained by an Interaction-driven model, emphasizing the synergy between individual agency and external support systems (e.g., organizational platforms, policy opportunities). A significant portion (35.24%) is attributed primarily to Internal-driven factors, such as exceptional personal effort and resilience. Notably, no cases were attributed solely to external factors.

To further contextualize these patterns, a binary logistic regression analysis of 1,219 recipients of the “China Youth May Fourth Medal” was conducted to examine the correlations between their growth factors and variables including award sessions, educational background, age, region, industry, and growth trajectories. Statistically significant relationships were identified, reflecting broader trends and regularities in the growth of outstanding contemporary youth groups. Specifically, two dimensions of correlations emerged:

As shown in Table 3: First, general correlations were observed between award sessions, industry, region, educational background, and growth factors. For instance, with the progression of selection years, the proportion of Internal-driven growth factors showed a slight upward trend, with average proportions of 23.50%, 25.05%, 27.68%, 25.14%, and 35.27% in the 1st–5th, 6th–10th, 11th–15th, 16th–20th, and 21st–26th sessions, respectively. In terms of industry, groups such as those in Administrative and Public Services and Science, Technology and Energy predominantly relied on Interaction-driven growth factors, while recipients in Industrial Services, Sports and Athletics, and other industries were more likely to be characterized by Internal-driven factors. Regionally, the proportion of Interaction-driven factors reached 83.50% among outstanding youth in Beijing, compared to 56.45% in Hubei. Additionally, higher educational attainment was associated with a greater tendency for outstanding youth to leverage team advantages, information technology, and policy support—an observation consistent with the societal trend of rising overall educational levels.

**TABLE 3:** Bivariate logistic regression analysis of growth factors of 1,219 China Youth May Fourth Medal recipients.

Classify		Time	Gender	Industry	Region	Educational background	Age	Growth trajectories
Growth factors	Conspicuousness	0.987	0.545	1.046	0.970	1.279	1.141	0.008
	Exp (B)	0.048	0.564	0.027	0.041	0.048	<0.001	<0.001

Second, significant correlations were found between age, growth trajectories, and growth factors. This means that the older recipients were when awarded, the more prominent Interaction-driven factors became in their growth processes, while Internal-driven factors were more salient in those with Inverted-ladder growth trajectories. For example, the proportion of Interaction-driven factors was 78.96% among recipients aged 30 and above, 27.91% among those aged 20–30, and 2.13% among those under 20 (e.g., athletes and students). Regarding growth trajectories, the proportion of Internal-driven factors was 1.65% in Ladder-shaped trajectories, 84.04% in Folded-ladder trajectories, and 96.10% in Inverted-ladder trajectories.

## 5 Discussion

### 5.1 Interpretation of key findings within the chinese context

The findings present a nuanced picture of PYD in China. The trend towards gender equilibrium and professional diversity aligns with national policies promoting equity and comprehensive development. However, the absence of pronounced “youthification” or an absolute “high-education” bias in awardees suggests a distinctive feature of the Chinese model: it seeks to recognize substantive contribution across a broad spectrum of society, thereby mitigating a purely credential-based elitism and potential class solidification.

The identified growth trajector—Ladder-shaped, Folded-ladder, and Inverted-ladder—demonstrate that there is no single pathway to exemplary achievement in China. This diversity reflects the complex interplay between individual agency and the opportunity structures provided by national strategies (e.g., rural revitalization, innovation-driven development). The predominance of the Interaction-driven factor (64.76%) robustly supports the core premise of our analytical framework: that exemplary development in China is a co-constructive process between the motivated individual and a supportive, state-facilitated environment. The spiritual qualities, particularly the emphasis on Dedication, Innovation, and Patriotism, represent a concrete fusion of universal PYD virtues (e.g., Character, Competence, Contribution) with the specific ideological and cultural expectations of the Chinese context—namely, the integration of the “small self” into the “larger self” of national development.

However, a discussion on the sustainability of exemplary development must contend with the stark counter-examples of the few awardees whose medals were later revoked due to serious violations of law or discipline (e.g., Wang Like, Zhou Mengbo, Ding Xuefu). A representative case is Wang Like, who received the medal at age 40. His career subsequently followed a prototypical “Ladder-shaped” trajectory of rapid advancement, rising from section chief to provincial-ministerial leadership. Yet, this very path, initially celebrated as a model of alignment between individual merit and state opportunity, culminated in severe criminal conduct. From a socio-legal perspective, this paradox reveals a critical tension within the award system as the key institutional mechanism that operationalizes the “strategic alignment.” While it successfully transforms macro-level policies into recognizable scripts for individual achievement, the very institutional recognition that cements this alignment may carry perverse incentives if not coupled with robust mechanisms for ongoing oversight and correction. The “halo effect” of national honor can insulate individuals from routine scrutiny, accelerated empowerment may outpace the capacity of personal character, and the path dependency created by the “model” identity can stifle necessary self-reflection. These cases of “de-exemplification” do not negate the mechanism’s explanatory power but critically expose its boundary conditions, underscoring the socio-legal imperative for systems that actively support long-term integrity alongside the celebration of early achievement.

### 5.2 Theoretical and practical implications

Theoretically, this study contributes to the PYD literature by illustrating how the universal principles of positive development are uniquely mediated by a specific socio-political and cultural context. The Chinese case, with its emphasis on collective goals and state-led opportunity structures, suggests that the “C” of Connection may carry a more profound meaning, encompassing a deep, purposeful linkage to national aspirations. This linkage is not merely psychological but is actively forged and reinforced through the socio-legal apparatus of state recognition. It challenges the predominantly individualistic assumptions of Western PYD models and proposes a more collectivist-oriented understanding of thriving.

Practically, the findings offer valuable insights for youth policy and education. The revealed correlation between awardee share and population size, alongside notable deviations, suggests that nomination mechanisms could be further calibrated to align more closely with provincial demographic bases while incorporating a compensatory coefficient for less developed regions. This would make the recognition ecosystem more equitable and responsive to the nation’s diverse talent geography. The typology of growth trajectories provides a framework for designing more personalized and effective youth development programs that cater to diverse pathways. Given the weak correlation between awards and regional economic resources, such programs should explicitly institutionalize regionally adaptive evaluation standards, valuing context-specific contributions like grassroots service, rural innovation, and local community leadership alongside traditional achievement metrics. For young individuals, the stories of awardees, especially those with Folded-ladder or Inverted-ladder trajectories, offer powerful counter-narratives to linear success models, emphasizing resilience, adaptability, and the value of contribution in diverse fields.

## 6 Conclusions

### 6.1 The core mechanism: strategic alignment between individual and nation

This study, through a systematic analysis of 1,219 China Youth May Fourth Medal recipients, reveals that exemplary growth in the Chinese context is best understood as a process of “strategic alignment”. It is not merely a product of individual striving or state design, but a dynamic process whereby personal agency proactively aligns with the “zones of valued contribution” continually defined and opened up by national development strategies within a specific legal and policy framework. The state-sponsored recognition system selects and amplifies these “aligned exemplars,” thereby legitimizing strategic priorities and motivating wider emulation. This mechanism explains the predominance of the interaction-driven model (64.76%) and crystallizes how personal achievement and national mission are synergistically forged in the recognized elite youth through the mediating force of institutionalized recognition.

### 6.2 Limitations and future directions

This study has several limitations. First, its reliance on publicly available media reports provides episodic snapshots rather than a continuous, dynamic view of developmental processes. Second, while the elite sample of national award recipients effectively illuminates the state-endorsed archetype of excellence, it cannot represent the experiences of the broader, non-recognized youth population in China, thereby limiting the generalizability of the findings. This focus also constrains direct cross-cultural comparability with Western PYD studies that often draw on more general youth populations.

To address these gaps and advance from descriptive correlation toward stronger causal inference, future research should adopt more robust designs. First, a matched case-control study is recommended, comparing national-level awardees with provincially recognized youth (matched on key demographics) to isolate the specific factors that propel individuals from regional to national exemplar status. Second, a longitudinal design is crucial, combining prospective cohort tracking of recent awardees over 3–5 years with a retrospective analysis of the rare “de-exemplified” cases (where honors were revoked). This dual approach can critically assess the sustainability of model behavior, the award’s long-term impact, and systemic risks. Finally, extending the inquiry to non-awardee youth cohorts and conducting comparative studies with exemplary youth in other cultural settings are essential for testing the generalizability of the identified pathways and refining our understanding of the cultural specificity and universality of PYD models.

## Data Availability

The original contributions presented in this study are included in this article/supplementary materials, further inquiries can be directed to the corresponding author.

## References

[B1] Bandura A. (1977). *Social Learning Theory.* Englewood Cliffs, NJ: Prentice Hall.

[B2] Benson P. L. Scales P. C. Syvertsen A. K. (2011). The contribution of the developmental assets framework to positive youth development theory and practice. *Adv. Child Dev. Behav.* 41 197–230. 10.1016/B978-0-12-386492-5.00008-7 23259193

[B3] Bernardi L. Huinink J. Settersten R. A. Jr. (2019). The life course cube: A tool for studying lives. *Adv. Life Course Res.* 41:100258. 10.1016/j.alcr.2018.11.004 36738031

[B4] Bhattacharya A. Mehrotra S. (2013). The journey of personal growth: A qualitative exploration of personal growth processes in young adulthood. *Psychol. Stud.* 58 456–463. 10.1007/s12646-013-0222-x

[B5] Bjørk R. (2019). The age at which noble prize research is conducted. *Scientometrics* 119 931–939. 10.1007/s11192-019-03065-4

[B6] Bozeman B. Dietz J. Gaughan M. (2001). Scientific and technical human capital: An alternative model for research evaluation. *Int. J. Technol. Manage.* 22 716–740. 10.1504/IJTM.2001.002988 35009967

[B7] Bucchi M. (2018). The winner takes it all?” Nobel laureates and the public image of science. *Public Understand. Sci.* 27 390–396. 10.1177/0963662518764948 29720064

[B8] Buenconsejo J. U. Ferrer-Wreder L. Dimitrova R. Pavlova I. Bosnar K. Bartoluci S. et al. (2025). Global profiles of positive youth development: A person-oriented analysis among emerging adults living in 21 countries. *J. Youth Adolesc.* 54 2094–2119. 10.1007/s10964-025-02174-z 40205131

[B9] Barbee A. P. Cunningham M. R. Winstead B. A. Derlega V. J. Gulley M. R. Yankeelov P. A. et al. (1993). Effects of gender role expectations on the social support process. *J. Soc. Issues* 49 175–190. 10.1111/j.1540-4560.1993.tb01175.x

[B10] Chan K. S. Lai J. T. Li T. (2022). Cultural values, genes and savings behavior in China. *Int. Rev. Econ. Finance* 80 134–146. 10.1016/j.iref.2022.02.009

[B11] Coleman J. S. Campbell E. Q. Hobson C. J. McPartland J. Mood A. M. Weinfeld F. D. et al. (1966). *Equality of Educational Opportunity.* Washington, DC: US Government Printing Office.

[B12] Damon W. (2004). What is positive youth development? *Ann. Am. Acad. Polit. Soc. Sci.* 591 13–24. 10.1177/0002716203260092

[B13] Dowling G. R. (1986). Managing your corporate images. *Ind. Mark. Manage.* 15 109–115. 10.1016/0019-8501(86)90051-9

[B14] Gehmlich K. Krause S. (2024). Gender distribution of scientific prizes is associated with naming of awards after men, women or neutral. *Data* 9:84. 10.3390/data9070084

[B15] General Office of the Communist Party of China (CPC) Central Committee, & General Office of the State Council of the People’s Republic of China,. (2023). *Several Measures on Further Strengthening the Cultivation and Utilization of Youth Science and Technology Talents.* Available online at: https://www.gov.cn/zhengce/202308/content_6900456.htm (accessed January 18, 2025).

[B16] Han Q. Deng C. (2025). Evaluating the development of China’s modern industrial system. *Finance Res. Lett.* 74:106676. 10.1016/j.frl.2024.106676

[B17] Hitlin S. Piliavin J. A. (2004). Values: Reviving a dormant concept. *Annu. Rev. Sociol.* 30 359–393. 10.1146/annurev.soc.30.012703.110640

[B18] Hofstede G. (1991). *Cultures and Organizations: Software of the Mind.* London: McGraw-Hill International.

[B19] Huang B. Tani M. Wei Y. Zhu Y. (2022). Returns to education in China: Evidence from the great higher education expansion. *China Econ. Rev.* 74:101804. 10.1016/j.chieco.2022.101804

[B20] Kennedy S. H. (1977). Nurturing corporate images. *Eur. J. Mark.* 11 119–164. 10.1108/EUM0000000005007

[B21] Kyvik S. (1990). Age and scientific productivity: Differences between fields of learning. *High. Educ.* 19 37–55. 10.1007/BF00142022

[B22] Lerner R. M. Almerigi J. B. Theokas C. Lerner J. V. (2005). Positive youth development a view of the issues. *J. Early Adolesc.* 25 10–16. 10.1177/0272431604273211

[B23] Lerner R. M. Boyd M. J. Kiely M. K. Napolitano C. M. Schmid K. L. Steinberg L. (2011). The history of the study of adolescence. *Encycl. Adolesc.* 1 169–176. 10.1016/B978-0-12-373951-3.00019-3

[B24] Lerner R. M. Dowling E. M. Anderson P. M. (2003). Positive youth development: Thriving as the basis of personhood and civil society. *Appl. Dev. Sci.* 7. 10.1207/S1532480XADS0703_8 12448284

[B25] Li J. Zhao Y. Lin L. Chen J. Wang S. (2018). The freedom to persist: Belief in free will predicts perseverance for long-term goals among Chinese adolescents. *Pers. Individ. Dif.* 121 7–10. 10.1016/j.paid.2017.09.011

[B26] Li X. (2020). Investigating youth policies through the Lens of public narratives - Comparing China and Europe. *J. Youth Stud.* 24 614–633. 10.1080/13676261.2020.1751809

[B27] Li Y. Ji Q. Zhang D. (2020). Technological catching up and innovation policies in China: What is behind this largely successful story? *Technol. Forecast. Soc. Change* 153:119918. 10.1016/j.techfore.2020.119918

[B28] Liu A. P. L. (1984). Opinions and attitudes of youth in the People’s Republic of China. *Asian Surv.* 24 975–996. 10.2307/2644079

[B29] Liu X. Y. Jin N. (2019). Re-orientation of the policy of robbing people to fight in city–an analysis of focusing on young floating talents. *Chin. Youth Stud.* 2019, 47–53. 10.19633/j.cnki.11-2579/d.2019.0141

[B30] Longo A. F. Siffredi C. R. Cardey M. L. Aquilino G. D. Lentini N. A. (2016). Age of peak performance in Olympic sports: A comparative research among disciplines. *J. Hum. Sport Exerc.* 11 31–41. 10.14198/jhse.2016.111.03

[B31] Mahoney J. L. Harris A. L. Eccles J. S. (2006). Organized activity participation, positive youth development, and the over-scheduling hypothesis. *Soc. Policy Rep.* 20 1–32. 10.1002/j.2379-3988.2006.tb00049.x

[B32] Nam K.-M. (2017). Is spatial distribution of China’s population excessively unequal? A cross-country comparison. *Ann. Reg. Sci.* 60 700–723. 10.1007/s00168-017-0839-0

[B33] Overton W. F. (2013). A new paradigm for developmental science: Relationism and relational-developmental systems. *Appl. Dev. Sci.* 17 94–107. 10.1080/10888691.2013.778717 23834001

[B34] Rahkamo S. (2016). *The Road to Exceptional Expertise: Success Factors and Creative Processes of Five Multiple Olympic Gold Medallists*, Ph. D. dissertation. Espoo: Aalto University.

[B35] Roth J. L. Brooks-Gunn J. (2003). Youth development programs: Risk, prevention and policy. *J. Adolesc. Health* 32. 10.1016/S1054-139X(02)00421-4 12606110

[B36] Sakamoto H. (2010). Provincial economic growth and industrial structure in China: An index approach. *Ann. Reg. Sci.* 45 91–112. 10.1111/j.1757-7802.2011.01046

[B37] Scales P. C. Roehlkepartain E. C. Shramko M. (2017). Aligning youth development theory, measurement, and practice across cultures and contexts: Lessons from use of the Developmental Assets Profile. *Child Indic. Res.* 10 1145–1178.

[B38] Schunk D. H. (1987). Peer models and children’s behavioral change. *Rev. Educ. Res.* 57 149–174. 10.2307/1170234

[B39] Shang H. P. Liu J. T. (2024). Policy effect and spatial differentiation of Beijing-Tianjin-Hebei coordinated development. *Acta Geograph. Sin.* 79 2020–2041. 10.11821/dlxb202408008

[B40] Shek D. T. L. Leung J. T. Y. Tan L. (2023). Social policies and theories on quality of life under COVID-19: In search of the missing links. *Appl. Res. Qual. Life* 18 1149–1165. 10.1007/s11482-023-10147-2 36855587 PMC9950016

[B41] Shin S. Y. (2022). News media image: A typology of audience perspectives. *J. Commun. Monogr.* 24 80–140. 10.1177/15226379221092019

[B42] Tedeschi R. G. Calhoun L. G. (1996). The posttraumatic growth inventory: Measuring the positive legacy of trauma. *J. Trauma. Stress* 9 455–471. 10.1002/jts.2490090305 8827649

[B43] Triandis H. C. (1995). *Individualism and Collectivism.* Boulder, CO: Westview Press.

[B44] Wang Y. Tan G. Zhu T. (2025). The changes, formation and public policy measures of mental health in Chinese youth. *Front. Psychol.* 16:1583594. 10.3389/fpsyg.2025.1583594 40313905 PMC12044530

[B45] Zhang Y. Zhang Q. Lu Q.-B. Dong Q.-Y. (2024). Patterns in the evolution of China’s volunteering policy: A bibliometric analysis of policy documents from 1978 to 2019. *Chin. Law Gov.* 52 57–83. 10.1080/00094609.2023.2285592

[B46] Zuckerman H. (1996/1977). *Scientific Elite: Nobel Laureates in the United States.* New Brunswick, NJ: Transactions.

